# Simulation of gas sensing with a triboelectric nanogenerator

**DOI:** 10.3762/bjnano.12.41

**Published:** 2021-05-28

**Authors:** Kaiqin Zhao, Hua Gan, Huan Li, Ziyu Liu, Zhiyuan Zhu

**Affiliations:** 1 School of Microelectronics, Fudan University, Shanghai 200433, China; 2 Chongqing Key Laboratory of Nonlinear Circuits and Intelligent Information Processing, Southwest University, Chongqing 400715, China; 3 No.29 Research Institute of CETC, Chengdu 610036, China; 4 Ocean College, Zhejiang University, Zhejiang 316021, China

**Keywords:** gas, sensor, triboelectric nanogenerator (TENG)

## Abstract

Safety concerns require the frequently check for leaks in gas pipelines. Also, in coal mines the type gases permeating from the ground need to be monitored. Triboelectric nanogenerators (TENGs) can be applied for gas sensing without external power supply. In this paper, a two-dimensional model of a TENG was established, and a gas jet a rectangular cross section was added between two triboelectric materials. The TENG could generate distinguishable electrical signals according to the different types of gas and the different gas injection areas. This work contributes to the area of self-powered gas sensing.

## Introduction

With economic development and social progress, there is an increasing demand for wearable [1–4], medical [5], and portable electronic devices [6–7]. The power supply is an important part of modern electronic equipment. A traditional battery has the disadvantages of short work life and heavy pollution. Therefore, it is extremely urgent to find a green and sustainable power supply for microelectronic devices.

Triboelectric nanogenerators (TENGs) can collect and convert different forms of energy (e.g., human motion [8–10], vibration [11], rotation [12], wind [13], and water [14]) into electric energy [15–17], thus expanding the range of energy production to a more microscopic scale [18] and improving the rate of utilization [19]. Characterized by low cost, light weight, environmental safety, and high conversion efficiency under low frequency, TENGs can provide continuous power supply for wearable devices [20], medical devices, and microelectronic systems [21]. In addition, triboelectric nanogenerators can also be used as sensors [22]. TENGs, originally proposed by Prof. Zhongling Wang [23], are microgenerators that convert mechanical energy into electrical energy based on the triboelectric effect [24]. In most TENG simulations, a triboelectric polymer material is in direct contact with an electrode layer with very small separation distance (less than 1 mm) [25], and the effective contact area of the friction material is increased by texturing its surface [26–27] to improve its electrical output. This setup is widely applied in vertical contact separation mode [28–29], sliding mode [30–31], single electrode mode [32–34], and independent layer mode [35]. In order to explain the charge transfer process between two friction materials in contact, various models have been proposed and explored, such as electron cloud model [36–38], ion transfer model [39], and material transfer model [40].

It is attractive that, in addition to providing power for electronic devices, TENGs can also be used as self-powered sensors for pressure, vibration, speed, chemicals, and body motion. Regarding leaks in gas pipelines or harmful gases in underground coal mines, it is necessary to detect the presence of a specific gas or the content of gas in ambient air. Therefore, gas sensors are usually indispensable in safety systems. Ordinary sensors need to be charged externally, and once the power is used up, the gas sensor loses its function. TENGs generate electricity that can be used for developing self-powered gas sensors.

In this paper, in order to explore the sensing of different gases by TENGs, a gas jet of rectangular cross section was added to the two-dimensional model of a TENG. The TENG generates electrical signals depending on the type of gas and the cross section of the gas injection area. Further, in order to eliminate the inevitable topological change during the actual movement of the TENG, an air gap was established in COMSOL to construct the two-dimensional model of the TENG. Based on the assumption that the surface charge density of the triboelectric nanogenerator is constant, the potential was calculated by using finite element methods and the Poisson equation of static electricity, and the influence of the change in the shape of the triboelectric material was simulated.

## Results and Discussion

**Figure 1:**
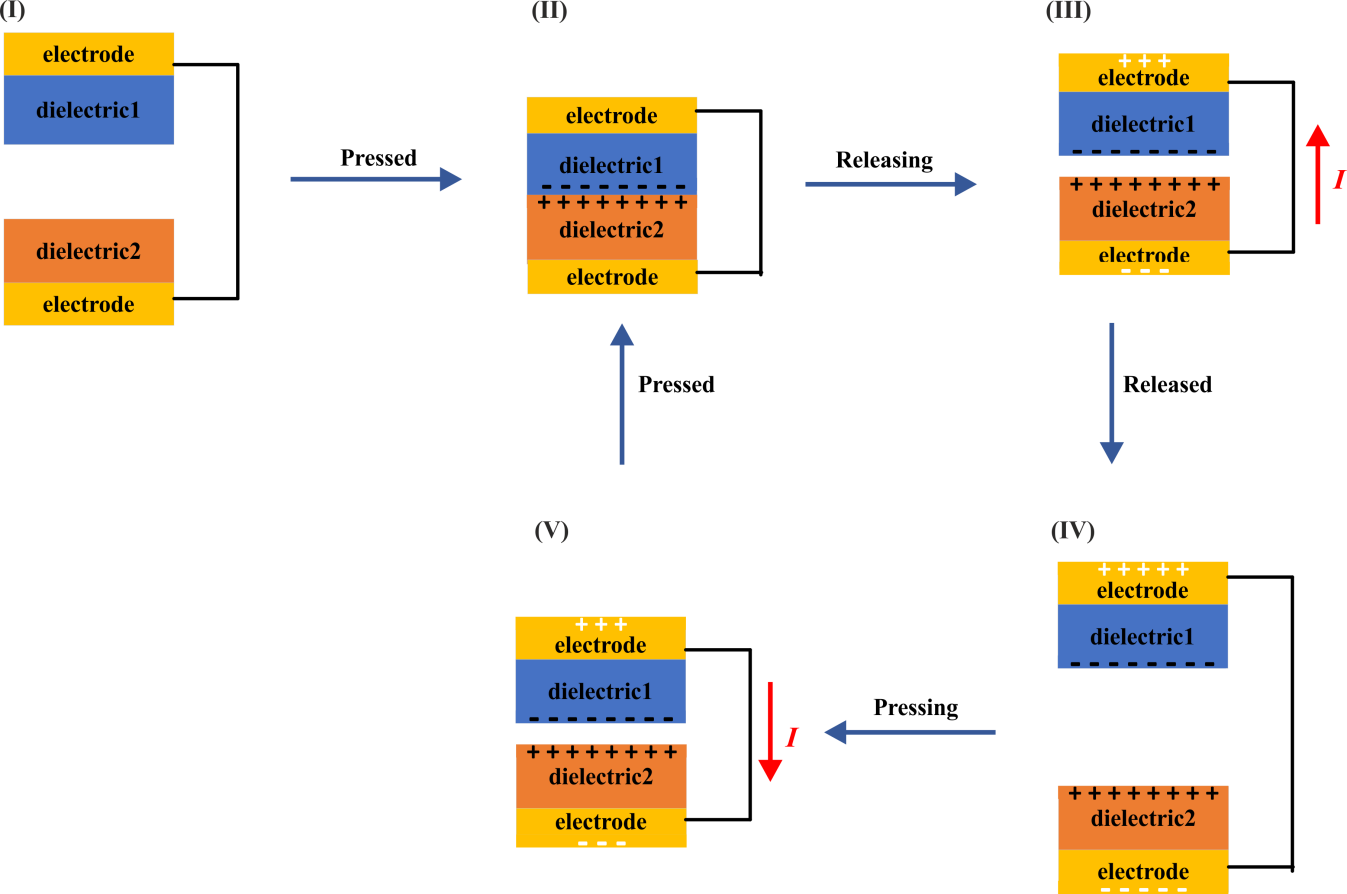
Charge flow in a triboelectric nanogenerator.

Triboelectric nanogenerators generate electricity through contact electrification and electrostatic induction. Contact electrification refers to the electron transfer between two different materials in contact because the atoms are so close together. An electric field is generated after friction electrification, and electrostatic induction is caused by the electric field. The charge flow of a TENG is shown in Figure 1. When the two triboelectric materials contact each other, different charges are generated on the surface. When they are separated, the induced electrons of the upper surface electrode will flow to the lower surface electrode, forming a current flow. When the two triboelectric materials approach, the electrons of the electrode on the lower surface will flow back to the electrode on the upper surface, forming a downward current until the two triboelectric materials contact each other.

**Figure 2:**
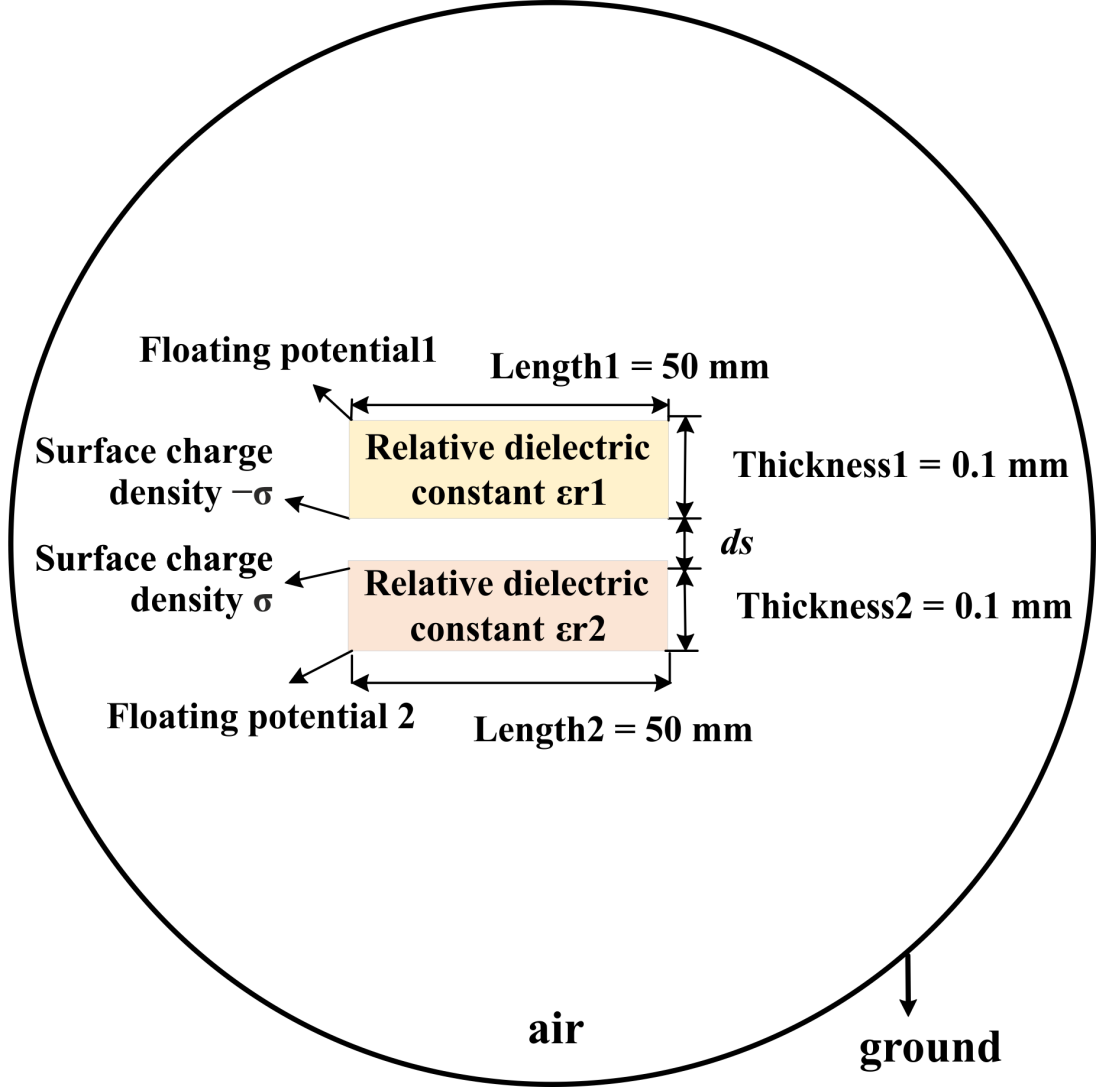
Two-dimensional model of a contact separation TENG.

**Figure 3:**
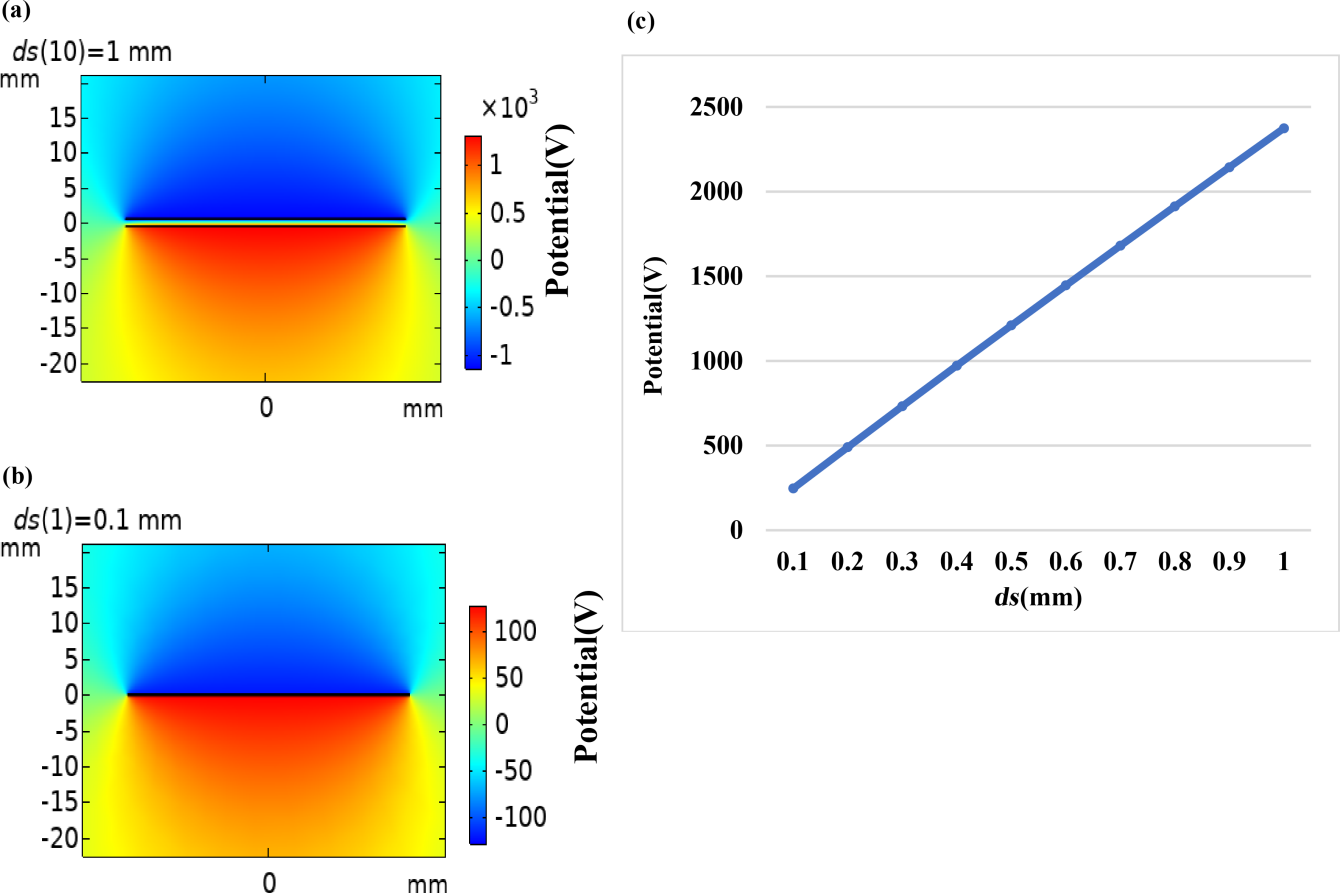
Simulation diagram for (a) *ds* = 1 mm and (b) *ds* = 0.1 mm. (c) Potential difference between the outer surfaces of the upper and the lower triboelectric materials as a function of *ds*.

A simplified model of a two-dimensional TENG was set up in COMSOL (Figure 2). Two rectangles represent the triboelectric materials with different dielectric constants. The length and width are set as 50 mm and 0.1 mm, respectively. By changing the distance (*ds*), we simulate the process of the triboelectric materials approaching and moving away from each other. Figure 3a is the surface potential distribution diagram of the triboelectric materials of a TENG at a distance of 1 mm. Due to the influence of the relative permittivity, the material with the lower relative permittivity is negatively charged, while the other triboelectric material is positively charged. When the distance between the two dielectric materials varies, the field intensity caused by the charge also varies. The corresponding electric potential decreases with decreasing distance and increases with increasing distance. Figure 3b is the electric potential distribution diagram when the distance is 0.1 mm. In the simulation, we measured the potential difference between the outer surfaces of the two dielectric materials as *ds* is gradually increased from 0.1 to 1 mm, as shown in Figure 3c, which also reflects the influence of the distance between the two triboelectric materials on the potential.

**Figure 4:**
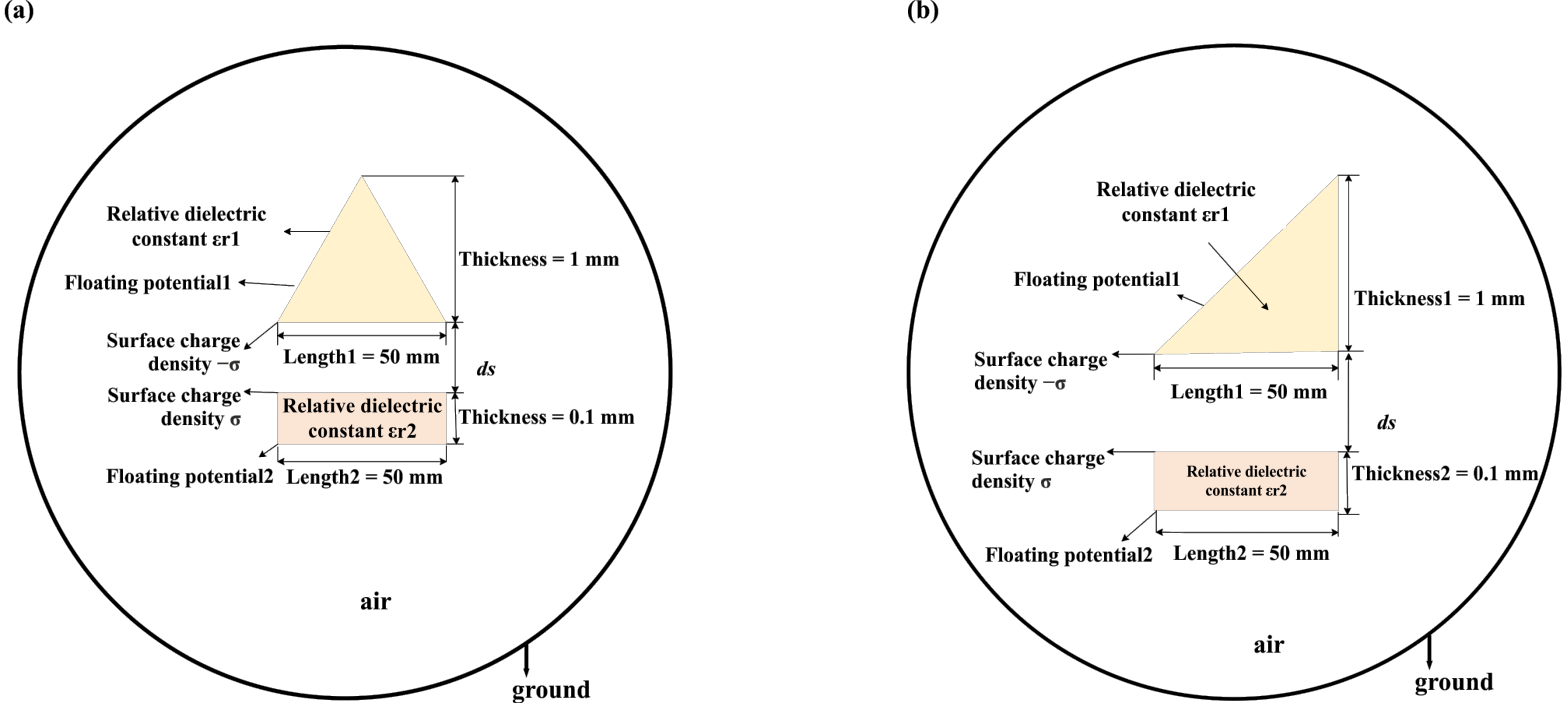
Two-dimensional TENG model with (a) isosceles triangle and (b) right-angled triangle TENG model.

In practice, it is difficult to directly test the influence of the shape of the triboelectric material on the electric potential due to the influence of various factors. Here, we set the shape of the upper triboelectric material to be an isosceles triangle and a right-angled triangle, the height is set to 1 mm, and the other parameters are the same as in Figure 2, as shown in Figure 4.

**Figure 5:**
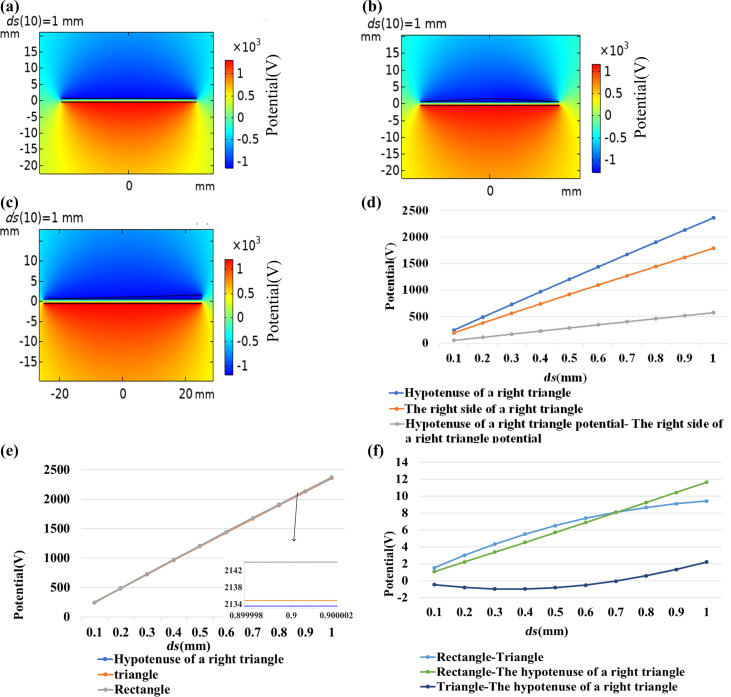
Comparison of shapes. (a) Potential distribution diagram of a rectangular TENG. (b) Potential distribution diagram of an isosceles triangle TENG. (c) Potential distribution diagram of a right-angled triangle TENG. (d) Outer boundary potential and potential difference of a right-angled triangle TENG. (e) Potential of the three shapes of TENG. (f) Potential difference of the three shapes of TENG.

The three models were simulated and compared in COMSOL while ignoring edge effects. As can be seen from Figure 5a–c, the electric potential of the rectangle and the isosceles triangles is symmetrically distributed along the central axis, while that of the right-angled triangle is asymmetrically distributed. Figure 5b shows that the two sides of the isosceles triangle have the same potential. According to Figure 5c, the potential distribution at the hypotenuse of the right-angled triangle is different from that at the side perpendicular to the horizontal plane. The potentials at the hypotenuse and the right side of the right-angled triangle were simulated for *ds* values of 0.1, 0.2, 0.3, 0.4, 0.5, 0.6, 0.7, 0.8, 0.9, and 1 mm, and the potential difference was calculated. Figure 5d shows that the electric potentials at the hypotenuse and the right side of the triangle increase with an increase of *ds*. Also, the potential difference increases. Therefore, if an external circuit can be built, then the load can be directly connected to the right side and the hypotenuse of the right-angled triangle and the transfer of electrons can be driven by the potential difference between the two sides. Figure 5e,f shows the electric potential in the simulation of rectangle, isosceles triangle, and right-angled triangle. The electric potentials obtained for the three models are basically the same, but it can be found that the electric potential calculated for the rectangular TENG is higher than those calculated for the triangles. Figure 5f is the potential difference obtained by subtracting the potential of the isosceles triangle TENG and that of the hypotenuse of the right-angled triangle TENG. The potential difference is all positive, and the potential difference increases as *ds* increases. The potential of the hypotenuse of the right-angled triangle TENG is smaller than that of the right-angled side, but as the distance *ds* increases, the gap between the potentials of the two sides decreases until the potential of the hypotenuse is greater than that of the right-angled side. It can be concluded that the rectangular TENG is economical and can achieve good electronic output.

**Figure 6:**
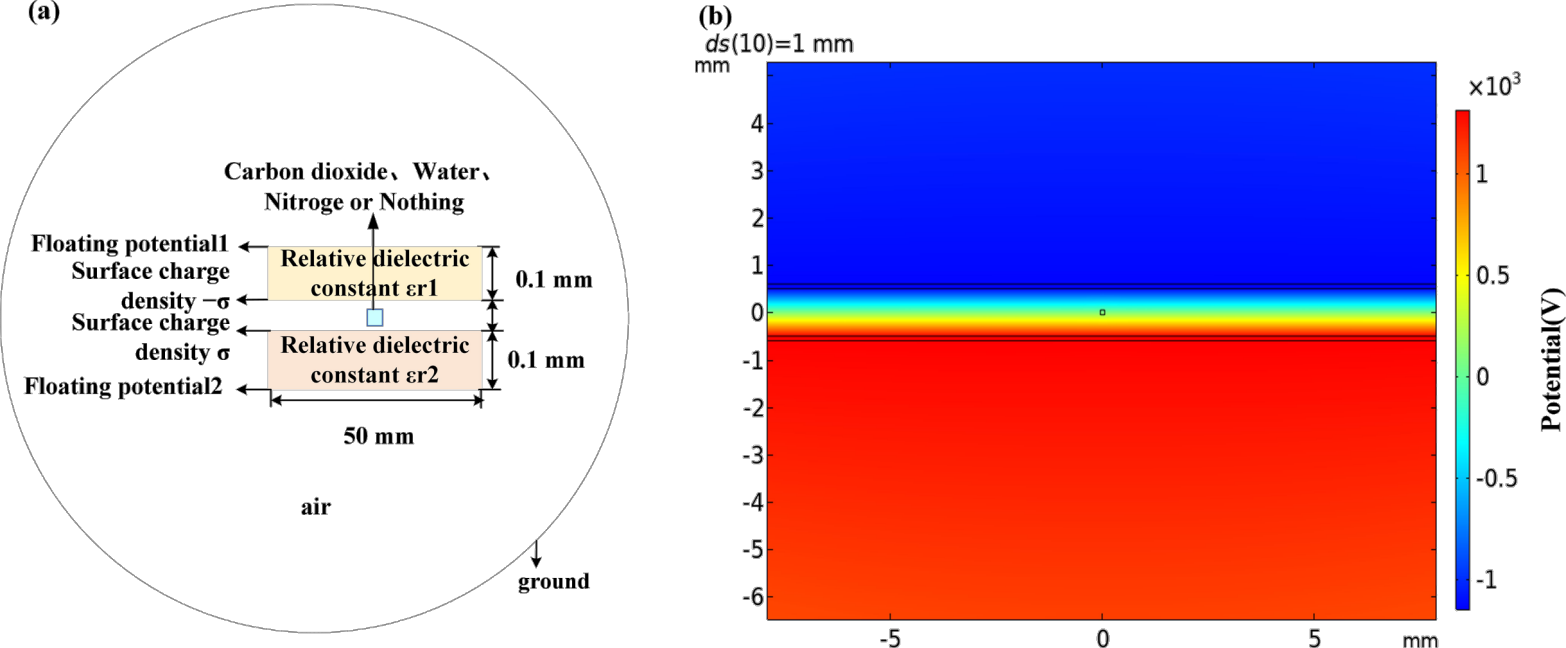
(a) TENG with a gas jet between two triboelectric materials. (b) TENG potential distribution diagram with gas jet.

We considered designing a TENG-based gas sensor that could be used to detect different gases under real-life conditions. When, in contact separation mode, two triboelectric materials approach or move away from each other, other substances, such as water vapor, carbon dioxide, and other gases, can pass through the gap. We present a simulation with water vapor under ideal conditions, that is, the surface charge density may be changed during the experiment. A simplified two-dimensional sensor model is shown in Figure 6a. A rectangular gas injection area was added to the model in Figure 2. It was used to study the influence of gas type and gas injection area on the electrical potential of the TENG. The potential distribution obtained from COMSOL is shown in Figure 6b.

**Figure 7:**
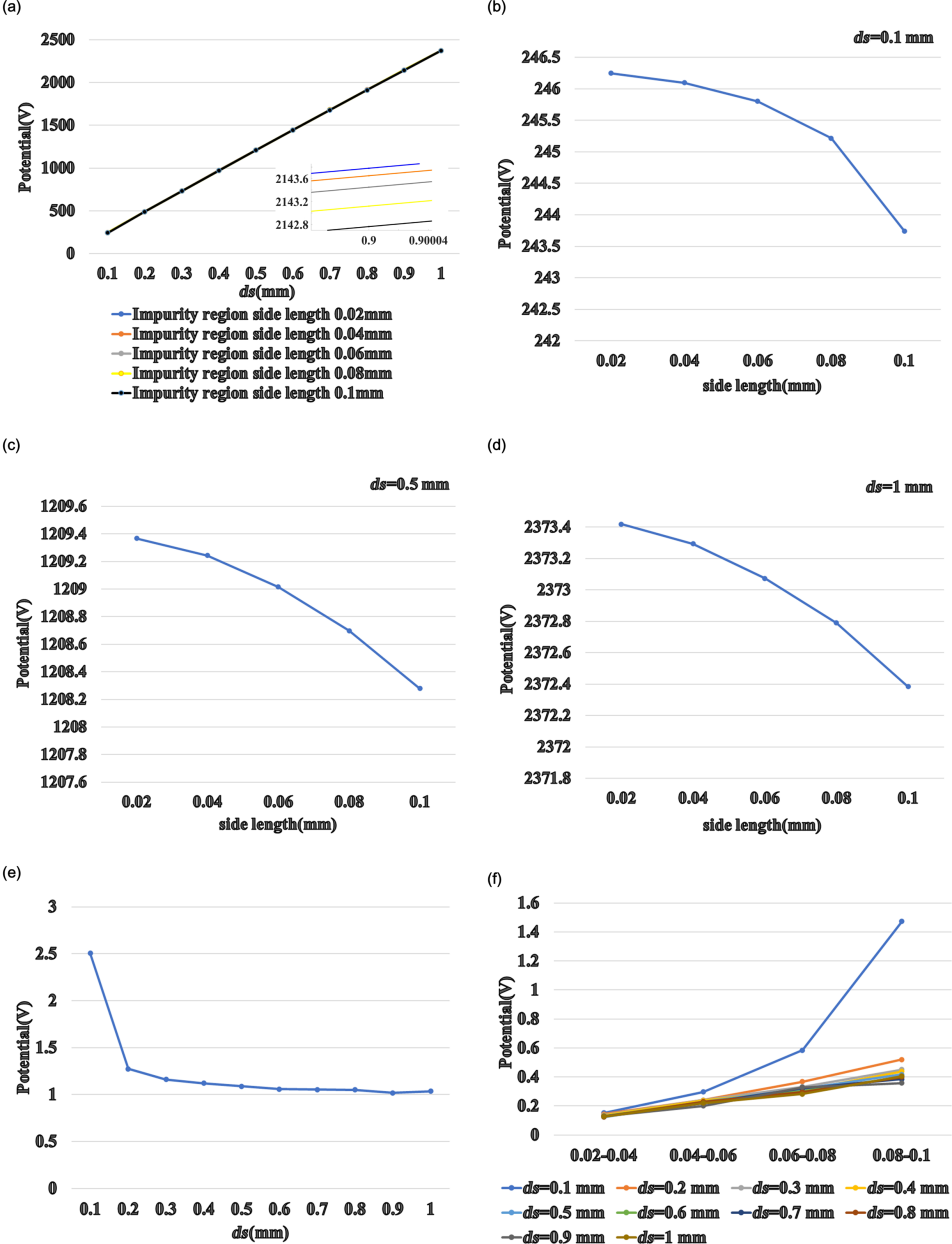
(a) The potential of the TENG varies with distance *ds* for different sizes of the gas injection area. The TENG potential as a function of the gas injection area with (b) *ds* = 0.1 mm, (c) *ds* = 0.5 mm, and (d) *ds* = 1 mm. (e) TENG potential difference as a function of *ds*. (f) TENG potential difference of rectangular cross sections with different side lengths.

The injection of carbon dioxide into the air gap of the TENG was simulated to quantitatively analyze the gas injection area. The effect on the TENG potential was first considered as a function of the gas injection area (indicated here by the side length of the rectangle) and the distance between the two triboelectric materials, as shown in Figure 7a. When gas injection area changes, the TENG potential also changes slightly. Figure 7b–d shows that, with constant distance between the two triboelectric materials, an increase in the size of the gas injection area leads to a decrease of the electric potential, suggesting that the TENG gas sensor can be used to measure the size of a gas leak.

Figure 7e shows the TENG potential as a function of the distance *ds*. For *ds* = 0.1 mm, the difference in TENG potential is the largest (approximately 2.5 V). When *ds* increases, the potential difference between a gas injection area of 0.02 mm and a gas injection area of 0.1 mm decreases, eventually reaching 1 V. This suggests that when the distance between the two triboelectric materials is large enough relative to the size of the gas injection area, the size of the gas injection area has little effect on the sensing function. Therefore, if the gas injection area needs to be measured, it should be first estimated, then an appropriate TENG distance *ds* needs to be found.

The potential differences of the TENG with a rectangular gas injection area with different side lengths are compared in Figure 7f. The curves are closer to a quadratic function when *ds* = 0.1 mm. Based on the knowledge of differential derivation in mathematics, Figure 7b can be speculated to be a cubic curve. The curves of the four potential differences are closer to linear curves for *ds* = 0.2–1.0 mm. Similarly, it can be inferred that Figure 7c and Figure 7d are curves of a quadratic function.

**Figure 8:**
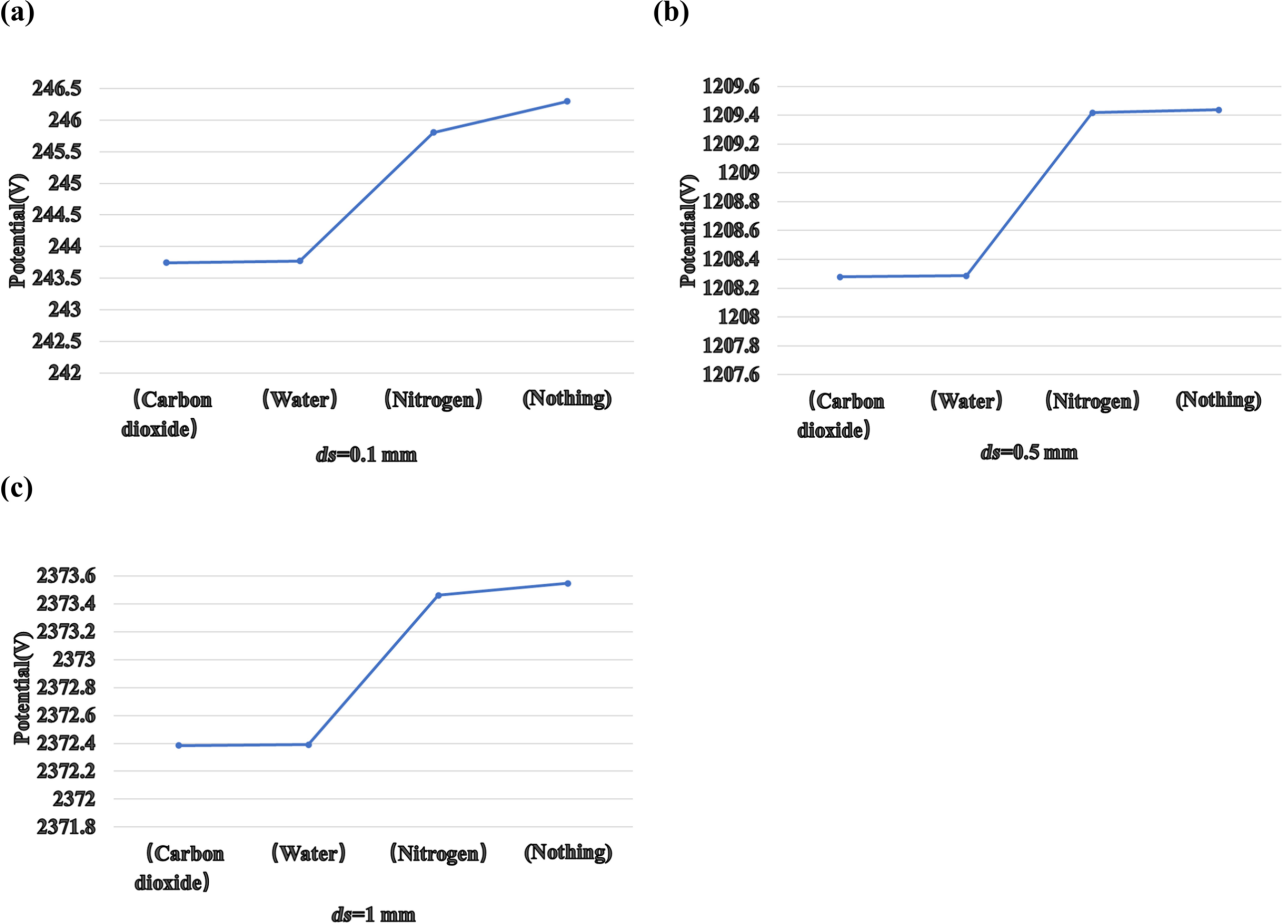
(a) The potential of TENG with different injected gases at (a) *ds* = 0.1 mm, (b) *ds* = 0.5 mm, and (c) *ds* = 1 mm.

We compared the TENG with carbon dioxide, water vapor, and nitrogen as injection gases. Figure 8 shows the potential difference of the TENG with different gases at the same distance *ds*. The potential values of the TENG with carbon dioxide and water vapor are similar. The potential of the TENG with nitrogen is significantly higher. Without any gas, the potential of the TENG is even higher. It is noted that the relative dielectric constants of these three gases are different. That of carbon dioxide is the largest, followed by those of water vapor and nitrogen, which explain the results shown in Figure 8. It can also be concluded from these figures that the difference in power potential between the four types of TENG decreases with the increase of *ds*.

**Figure 9:**
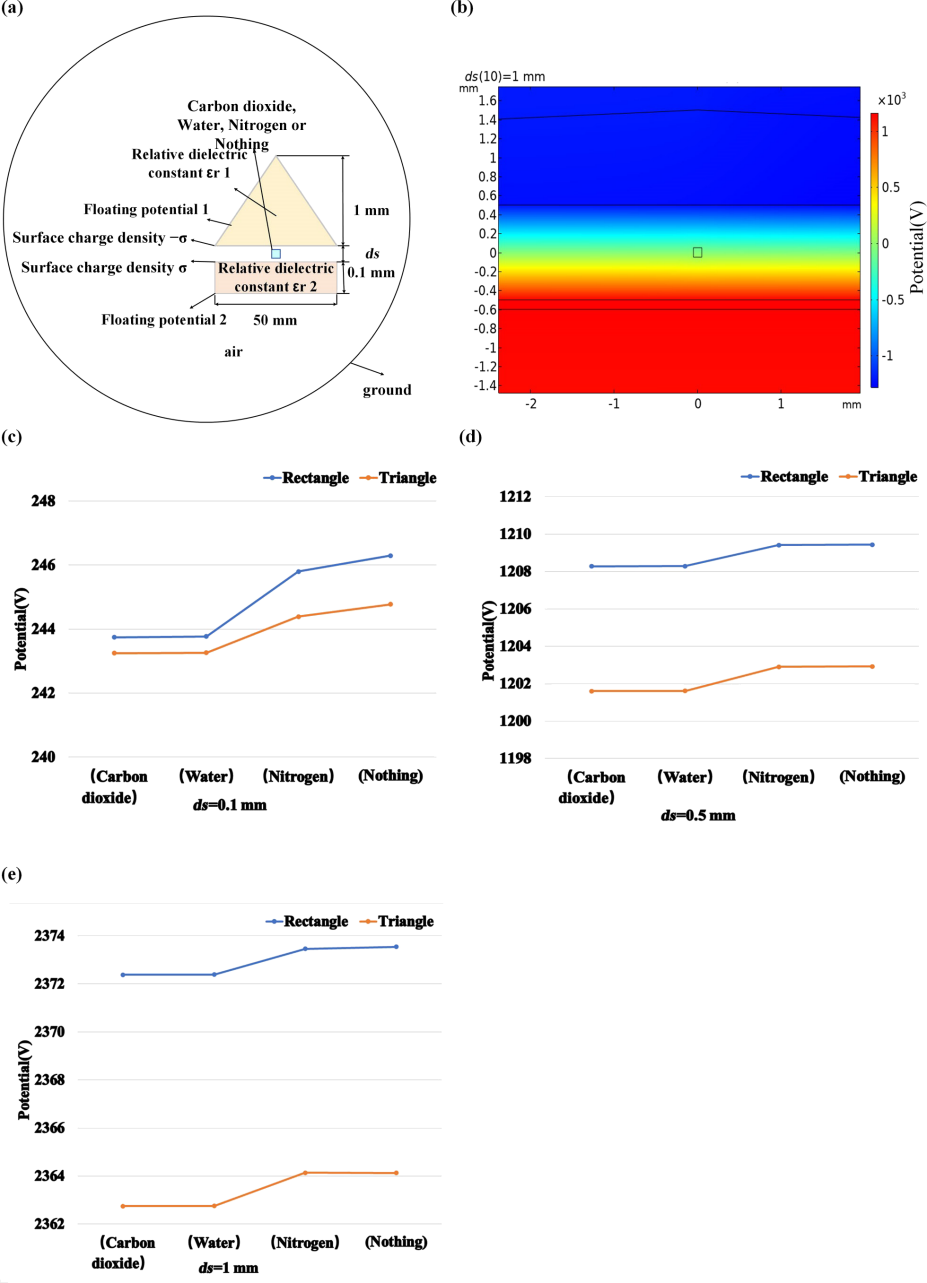
(a) Schematic diagram of an isosceles triangle TENG with injected gases. (b) Simulation of the TENG potential. (c–e) Potential diagram of the two shapes of TENGs with different injected gases at the same distance *ds*.

Figure 9a is the schematic diagram of an isosceles triangle TENG with a gas jet. Figure 9b is the corresponding simulation of the potential. In order to compare the effects of the two shapes of TENG on sensing different gases, we simulate the TENG with gas jets when *ds* is 0.1 mm, 0.5 mm, and 1 mm, as shown in Figure 9c–e. It can be seen from these three figures that a small distance between the triboelectric materials is helpful for distinguishing different gases. It is noted that water or humidity can degrade the surface charge density of the electrification surfaces in an actual experiment. We will carry out further investigations in the future.

## Conclusion

Simulations of three differently shaped TENGs were carried out in this paper. In order to investigate the sensing of different gases with sensors based on these TENGs, an injected gas jet with rectangular cross section was added to the two-dimensional model of the TENG. The size of the gas jet influences the potential of the TENG. However, when the distance between the triboelectric materials is large enough, a change of the gas jet cross section has only little effect on the TENG potential. The simulation results also show that the type of gas influences the potential of the TENG depending on the relative permittivity of the gas. This work is helpful for the development of self-powered TENG-bases gas sensors.
